# Anti-Mold Protection of Textile Surfaces with Cold Plasma Produced Biocidal Nanocoatings

**DOI:** 10.3390/ma15196834

**Published:** 2022-10-01

**Authors:** Ewa Tyczkowska-Sieroń, Agnieszka Kiryszewska-Jesionek, Ryszard Kapica, Jacek Tyczkowski

**Affiliations:** 1 Department of Experimental Physiology, Medical University of Lodz, Mazowiecka Str. 6/8, 92-215 Lodz, Poland; 2 Department of Microbiology and Laboratory Medical Immunology, Medical University of Lodz, Pomorska Str. 251, 92-213 Lodz, Poland; 3 Department of Molecular Engineering, Faculty of Process and Environmental Engineering, Lodz University of Technology, Wólczańska Str. 213, 93-005 Lodz, Poland

**Keywords:** textile fabric, antifungal protection, nanocoating, biocidal compounds, cold plasma, plasma-activated grafting, plasma polymerization

## Abstract

The permanent anti-mold protection of textile surfaces, particularly those utilized in the manufacture of outdoor sporting goods, is still an issue that requires cutting-edge solutions. This study attempts to obtain antifungal nanocoatings on four selected fabrics used in the production of high-mountain clothing and sleeping bags, and on PET foil as a model substrate, employing the cold plasma technique for this purpose. Three plasma treatment procedures were used to obtain such nanocoatings: plasma-activated graft copolymerization of a biocidal precursor, deposition of a thin-film matrix by plasma-activated graft copolymerization and anchoring biocidal molecules therein, and plasma polymerization of a biocidal precursor. The precursors used represented three important groups of antifungal agents: phenols, amines, and anchored compounds. SEM microscopy and FTIR-ATR spectrometry were used to characterize the produced nanocoatings. For testing antifungal properties, four species of common mold fungi were selected: *A. niger*, *A. fumigatus*, *A. tenuissima*, and *P. chrysogenum*. It was found that the relatively best nanocoating, both in terms of plasma process performance, durability, and anti-mold activity, is plasma polymerized 2-allylphenol. The obtained results confirm our belief that cold plasma technology is a great tool for modifying the surface of textiles to provide them with antifungal properties.

## 1. Introduction

The problem of mold growth on textile products is of particular concern in the case of sports and outdoor clothing, for example, mountain suits, as well as other types of equipment using fabrics, such as sleeping bags. Atmospheric conditions, especially high humidity and high temperature, and sweating, and hard to avoid soiling by organic substances that serve as nutrients for the fungi are the main reasons for their expansion. This undoubtedly affects the performance parameters of these products and also poses a threat to users.

For a long time, attempts have already been made to obtain the antimicrobial properties of fabrics and textile products [1], however generally focusing on pathogenic bacteria, viruses, and sometimes fungi (mainly of the genus *Candida*) [2,3,4,5], leaving somewhat out of the way common molds [6,7], which can also be hostile and dangerous.

The selection of appropriate biocides to obtain antimicrobial properties on a given surface is related to the mechanism of their interaction with microorganisms. From this point of view, biocides can be divided into four broad categories: (*1*) oxidants, which include agents containing, for example, chlorine or peroxides, which act directly through radical reactions with the organic material of the microbial cell; (*2*) electrophilic biocides, which include both inorganic agents containing metal ions, for example, silver or copper, and organic electrophiles, for example, formaldehyde, which inactivate enzymes and generate intracellular free radicals; (*3*) biocides that destabilize the cell membrane, such as amines, phenols, and alcohols, leading to rapid cell lysis; and finally, (*4*) protonophores, e.g., weak acids, which cause the proton motive force disturbance and as a result widespread disruption of metabolism in the cell interior [8,9,10].

On the other hand, from the point of view of biocide bonding on a textile surface, we can distinguish its permanent attachment (non-leaching type)—in this case, the microbe cell must approach the surface and come into direct contact with such a molecule—as well as the controlled release of the biocide to the surroundings (leaching type), which disrupts the microorganism in the vicinity of the textile surface, but may be less advantageous due to the systematic reduction of the antimicrobial surface activity and not always favorable environmental impact [9,11].

To date, a number of methods for the production of antimicrobial textile surfaces have already been developed, ranging from physically adsorbed active substances, through their coordination bonding to a properly functionalized surface, and ending with the strong covalent immobilization of biocides [12,13,14,15,16,17]. The latter procedure is of particular interest due to the high and long-lasting durability of such a modification.

A special place among the methods of surface modification leading to antimicrobial activity, also widely adapted by textiles, is occupied by the cold plasma technology, which offers great possibilities in this field [18,19]. Basically, this technology is used in three different ways: plasma-activated graft copolymerization, plasma surface functionalization, and thin-film plasma deposition [20]. The graft copolymerization involves the activation of the surface in the plasma of inert gas, as a result of which free radicals are created. They can be used as graft copolymerization centers by contacting (without plasma) the surface prepared in this way with an appropriate monomer, e.g., allylamine [21,22,23]. The antimicrobial agent molecules can then be anchored in the polymer “brush” thus produced, by intermolecular forces, for example, dipole–dipole interactions [22,23].

The surface functionalization, in turn, consists of introducing specific functional groups (e.g., hydroxyl and carboxyl) on the surface as a result of treatment with plasma of the appropriate chemical composition. Such groups can then be used for further chemical reactions forming covalent bonds (e.g., esterification and etherification) or hydrogen bonds (e.g., using hydroxyl groups on the surface) strongly binding the biocide molecules [24,25,26].

Antimicrobial agents can also be introduced on the textile surface in the form of a thin film, the structure of which we are able to control from the polymer-like form to highly cross-linked covalent glasses, depending on the plasma deposition parameters. Under mild deposition conditions, the molecular structure of the plasma process precursor is only partially decomposed, which on the one hand allows the properties of such a molecule to be preserved, for example, biocidal properties, and on the other hand, enables its chemical incorporation into the film structure. This procedure is commonly called plasma polymerization, although it is sometimes referred to as plasma-induced grafting, which must however be clearly distinguished from the aforementioned plasma-activated grafting [27,28]. The use of more drastic deposition conditions (e.g., high power plasma generation) leads to the significant fragmentation of the precursor molecules and the formation of a completely new material that serves as a matrix for the embedding of antimicrobial agents, most often as metal ions or nanoparticles (Ag and Zn) produced directly in the deposition process from organometallic precursors [29] or in a parallel plasma-sputtering process [30,31,32].

This work is part of a broader research program aimed at the comprehensive improvement of the properties of high-mountain equipment. Within the framework of the program, the production of superhydrophobic surfaces on fabrics [33,34], which are used to make down suits, jackets, and sleeping bags, as well as the production of superhydrophobic down [35,36], have already been developed using plasma methods. However, the problem of protecting the above-mentioned products against mold growth still remained to be solved. To obtain textile surfaces resistant to molds, cold plasma technology was also used in this case.

## 2. Materials and Methods

### 2.1. Textile Fabrics

Several commercially available fabrics delivered by Ripstop.pl (Poznań, Poland), which are used for producing high-mountain clothing, were selected for the research. These fabrics are summarized in Table 1. They include both synthetic (based on polyester or polyamide) and natural (cotton) materials. During the antifungal modification, the inner side of the fabrics was treated. Before the treatment, the fabrics were washed in an ultrasonic cleaner in ethyl alcohol 99.8% (Avantor Performance Materials Poland S.A., Gliwice, Poland) for 10 min and then in distilled water for 15 min, after which they were dried for 24 h at 30 °C. This procedure was aimed at removing various impurities (dust, fingerprints, etc.) from the surfaces and was inert to the fabrics used.

Polyethylene terephthalate (PET) foil (Plastics Group Sp. z o.o., Warsaw, Poland) with a thickness of 0.5 mm was used as a model material to study the course of surface modification processes and the chemical structure of the created antifungal nanocoatings.

### 2.2. Mold Fungi

Molds for research were isolated from a range of used and moldy down-filled clothes and sleeping bags that returned from high-mountain expeditions to the Himalayas and the Karakoram. The obtained microbial material was inoculated on Sabouraud liquid medium and solid medium with chloramphenicol and gentamicin (both from bioMérieux Polska, Warsaw, Poland). The cultures were incubated for 24 h at 37 °C and then 7–14 days at 25 °C. In this way, the mold fungi of the genus *Aspergillus*, *Penicillium*, *Alternaria*, and *Chaetomium* were isolated. Then, using the Czapek-Dox agar differentiation medium (Sigma-Aldrich, Poznań, Poland), individual species of fungi were identified. Within the genus *Aspergillus*, we found the species *A. niger*, *A. fumigatus*, and *A. flavus*, in turn, in the genus *Alternaria*—species *A. chlamydospora* and *A. tenuissima*, and in the genus *Penicillium*—species *P. chrysogenum* and *P. brevicompactum*. In this study, the species that were most frequently isolated by us, such as *A. niger*, *A. fumigatus*, *A. tenuissima*, and *P. chrysogenum*, were selected for further tests.

### 2.3. Antifungal Nanocoating Precursors

Considering the identified species of microorganisms and based on literature reports [8,9,10,12,37,38], appropriate chemical compounds with expected antifungal activity were selected. For plasma-activated grafting or plasma polymerization processes, these compounds, apart from the moiety in their structure responsible for potential antifungal properties (amine and phenol derivatives), had to have functional groups that readily participate in addition polymerization reactions (ethenyl or ethynyl groups). Antifungal compounds that can anchor into a properly prepared surface (without creating typical covalent bonds) were also used in these studies. The chemical compounds used in this work as precursors of antifungal nanocoatings are listed in Table 2. In the selection of precursors, their easy availability was also important (Merck KGaA, Darmstadt, Germany).

### 2.4. Preparation of Antifungal Nanocoatings

In each of the procedures used to prepare antifungal nanocoatings, cold plasma technology was employed. For this purpose, we adopted a parallel-plate plasma reactor operating at radio frequency (RF 13.56 MHz) under low pressure, which is described in more detail elsewhere [39]. When a sample (fabric or PET foil) was placed in the reactor chamber, it was evacuated down to approx. 0.1 Pa. Then, argon (99.999% purity, Linde Gas, Cracow, Poland) was introduced with a flow rate of 2.0 sccm and pressure inside the chamber of 7.0 Pa, followed by a glow discharge with a power of 40 W for 15–240 s. After the completion of this plasma treatment process (activation process), a suitable precursor was supplied to the reactor chamber to carry out a graft copolymerization process (without plasma) or a plasma polymerization process (involving plasma with significantly reduced power compared to that used in the argon plasma treatment).

In addition to the above nanocoatings covalently bonded to the substrate material, we also investigated nanocoatings obtained by anchoring antifungal molecules in a thin-film matrix formed on the textile surfaces. Plasma-activated allylamine graft copolymerization was used as the matrix. After the completion of the plasma treatment process, allylamine saturated vapor at room temperature (32.3 kPa) was introduced into the reactor chamber for 2 h. Then, the samples were placed in a 2% solution of the active substance (triclosan or 3,5-dichlorophenol) in ethyl alcohol (99.8%) for 24 h at 25 °C.

All prepared samples were washed with distilled water in an ultrasonic cleaner to remove unbound compounds and then dried at room temperature.

**Figure 1:**
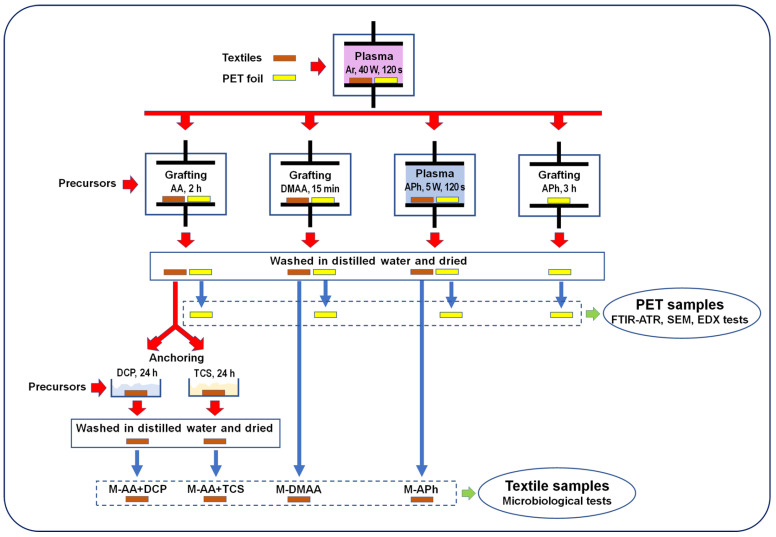
Diagram of the procedures for preparation of the tested in the work anti-mold nanocoatings and the investigations performed on them.

Detailed parameters of the nanocoating preparation processes are provided in the further part of the work when their individual cases are discussed. For clarity, a diagram of the procedures used in the work for the production of such coatings is shown in Figure 1.

### 2.5. Characteristics of Nanocoatings

To confirm the formation of nanocoatings by plasma-activated graft copolymerization or plasma polymerization processes, as well as to determine the rate of their deposition, Fourier transform infrared-attenuated total reflectance spectroscopy (FTIR-ATR) was employed. A Jasco FTIR 6200 spectrometer (JASCO Inter. Co., Ltd., Tokyo, Japan) equipped with an MCT detector cooled by liquid nitrogen (77 K) and a MIRacle^TM^ single reflection diamond ATR sampling accessory (PIKE Technol., Madison, WI, USA) was used for this purpose. The whole spectrometric system was purged by dry nitrogen (99.999% purity; Linde Gas, Cracow, Poland). FTIR-ATR spectra were recorded in the range of wavenumbers from 4000 to 600 cm^−1^, with a resolution of 1.0 cm^−1^ (300 scans) for model samples prepared from the nanocoatings on the PET foil. The as-recorded spectra were calibrated by reference to the PET foil spectrum. The interesting parts of the spectra were analyzed according to a numerical peak-fitting algorithm (PeakFit™, Systat Software Inc., San Jose, CA, USA).

The changes in the surface topography and quantitative analysis of elemental composition for samples of the textiles and PET foil before and after creating the nanocoatings were studied by scanning electron microscopy using a Quanta 200 F (FEI, Hillsboro, OR, USA) equipped with an X-max 50 EDS analyzer (Oxford Instruments, High Wycombe, UK). All measurements were carried out under a nitrogen atmosphere of 100 Pa (mode for non-conductive samples without sputter coating) and using the electron beam energy of 3.5 keV.

### 2.6. Microbiological Testing

The antifungal activity of the nanocoating precursors (Section 2.3) against the isolated species of mold fungi (Section 2.2) was determined by the dilution method. A suspension of the fungi in a water solution of 0.85% NaCl with a density of 0.5 on the McFarland scale (prepared based on preliminary investigations by the dilution method in the Sabouraud liquid medium) was evenly spread on Petri dishes with RPMI solid medium (bioMérieux Polska, Warsaw, Poland). After the suspension was completely absorbed by the medium (15 min), holes with a diameter of 8 mm were cut in it, and then 100 µL of the tested precursor solutions were introduced into the holes. The solutions were prepared in two concentrations of 0.01 and 0.1 mol/L by dissolving the appropriate amount of the respective precursor in a 10% dimethyl sulfoxide (DMSO) water solution. Then, the Petri dishes were incubated at 35 °C for 24–72 h, and after that time the diameter of the growth inhibition zones of the mold fungi tested was measured. All tests were repeated in triplicate. The measurement error did not exceed ±5%.

The evaluation of mold growth on the textile samples, both without and with antifungal nanocoatings, was carried out based on the Standard PN-EN 14119:2005 [40]. On each sample (3 × 3 cm in size), 0.5 mL of the Sabouraud agar was spread out and then allowed to dry, thus simulating the soiling of the textiles with nutrients for mold fungi and at the same time ensuring the same contact angle for all surfaces examined. On the samples prepared in this way, 0.1 mL of the selected fungi suspension with a density of 10^6^ cells/mL (density of 1 on the McFarland scale) was inoculated. The samples were incubated at 95% air humidity for 24 h at 37 °C, and then up to 14 days at 25 °C. The growth of the fungi on the test samples in the following days was visually assessed according to the scale from the above-mentioned standard:

0—No visible growth under the microscope (50×);
1—No visible growth to the naked eye, but clearly visible under the microscope;
2—Visible growth to the naked eye, coverage ≤ 25% of the area;
3—Visible growth to the naked eye, coverage ≤ 50% of the area;
4—Extensive growth, coverage > 50% of the area.

The results were taken as the average value of the same five samples.

## 3. Results and Discussion

### 3.1. Antifungal Activity of Precursors

**Figure 2:**
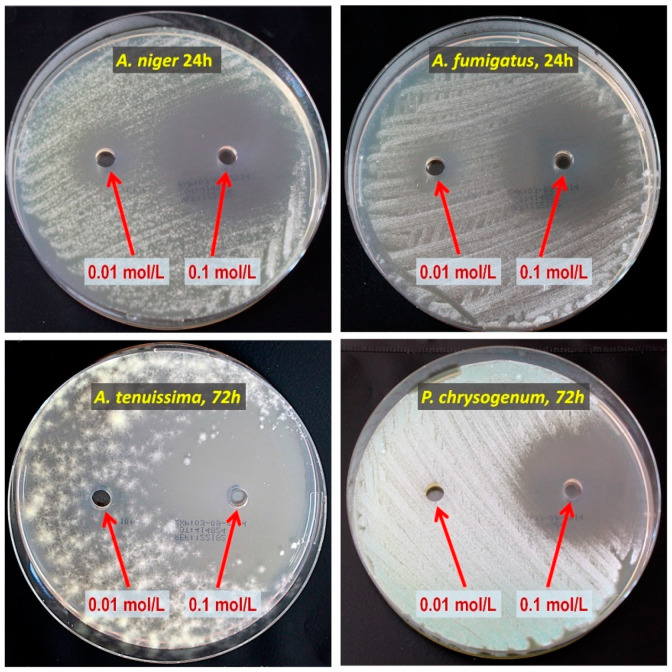
Example of a growth inhibition zone study for 2-allylphenol. The concentrations of the precursor solution, the mold fungi species, and the incubation period are provided in the photos.

The results of the study on the antifungal activity of the precursors from Table 2 against the designated species of mold fungi are presented in Table 3. In Figure 2, to illustrate the investigations, images of the growth inhibition zones for a few selected cases are shown.

Representatives from each group of precursors, namely phenols, amines, and anchored compounds, were selected for further study. High antifungal activity, according to Table 3, as well as their physicochemical properties, which are important for the preparation of nanocoatings, are taken into account here. For example, in the plasma-activated grafting and plasma polymerization processes, it is essential to have the boiling point (b.p.) of the precursor (see Table 2) as low as possible so that it can be easily vaporized. On the other hand, the anchoring molecules should have a strong interaction with the matrix to create the most stable system possible. Considering the above, further attention was paid to the following precursors: from phenols—2-allyphenol, from amines—*N*,*N*-dimethylallylamine, and from anchored compounds—3,5-dichlorphenol and triclosan. In addition, allylamine, which admittedly has no antifungal activity, has been used to produce a thin-film matrix with the anchored compounds.

### 3.2. Structure of Nanocoatings

Studies on the formation of nanocoatings from the selected precursors in the processes of plasma-activated grafting and plasma polymerization were carried out on model systems in which PET foil was used as a substrate. The main purpose of these studies was to prove the formation of nanocoatings, determine their basic structure, and select the most optimal conditions for their production for further applications on textile fabrics.

**Figure 3:**
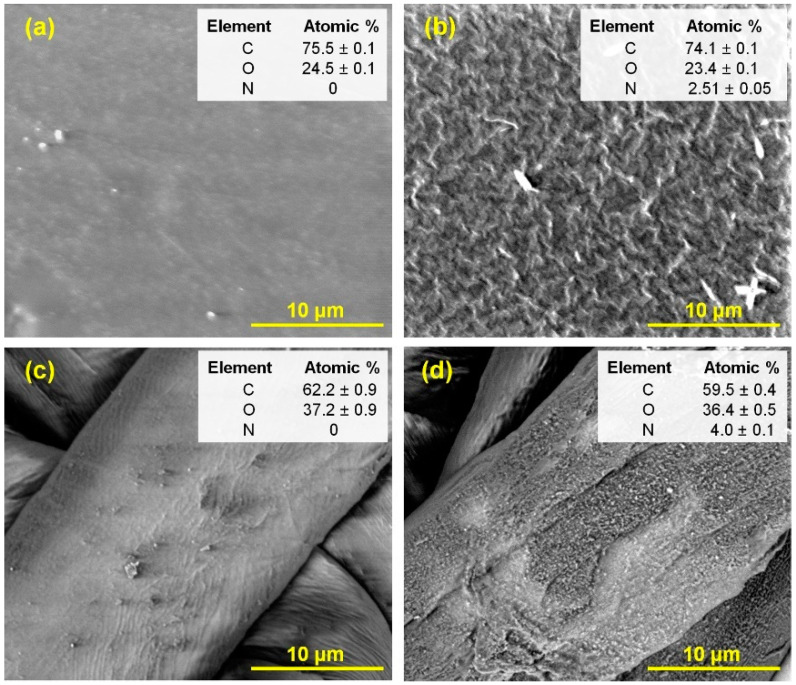
SEM images and EDS elemental composition analysis for: (**a**,**c**)—pure PET foil and T1 textile fiber, respectively; (**b**,**d**)—PET foil and T1 textile covered with plasma-activated allylamine grafting (Ar plasma activation at 40 W, 120 s; then graft copolymerization in saturated allylamine vapor at room temperature for 2 h).

The first precursor to be investigated, which we then used to form the thin-film matrix as the basis for anchoring antifungal molecules, was allylamine. Figure 3a,b shows the SEM images for pure PET and this foil after the plasma-activated allylamine grafting process. The evident changes in the surface topography may indicate the formation of a thin-layer coating on the PET foil. This is confirmed by the analysis of the elemental composition, which is also shown in this figure. Nitrogen is present in the sample after the plasma-activated grafting, but it is not found in pure PET, so it must originate from allylamine. Moreover, washing the sample in distilled water in an ultrasonic cleaner does not remove the deposited film, which proves its strong chemical bond with the PET substrate.

Additionally, in Figure 3c,d, we show SEM images of the textile fiber before and after plasma-activated allylamine grafting, together with the elemental composition. These results also confirm, similar to that of PET film, the formation of a nanocoating on the textile surface.

**Figure 4:**
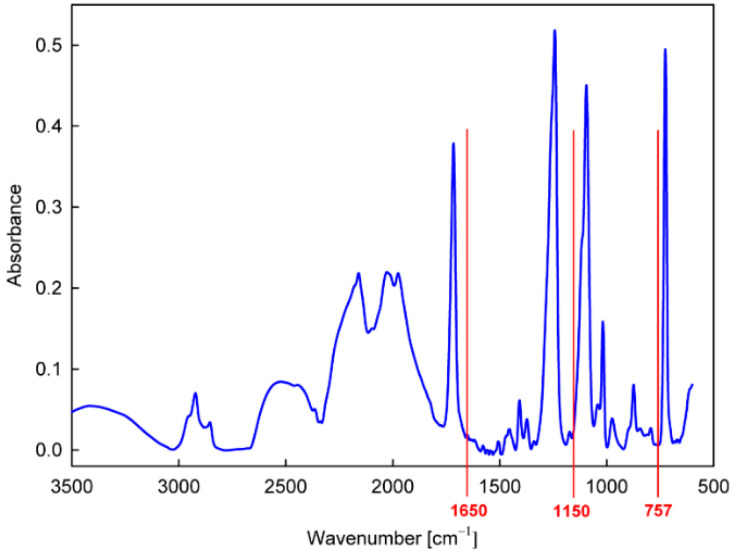
FTIR-ATR spectrum of the PET substrate.

**Figure 5:**
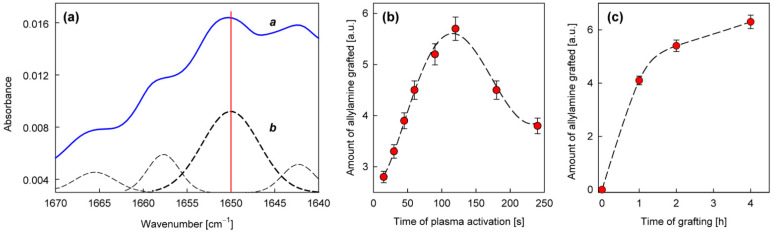
Characterization of the nanocoatings obtained from allylamine in plasma-activated grafting: (**a**)—An example of calibrated (*a*) and peak-fitted (*b*) FTIR-ATR spectrum in the region of 1650 cm^−1^ band (activation: Ar plasma, 40 W, 120 s; grafting: 1 h); (**b**,**c**)—The amount of allylamine grafted (calculated from the 1650 cm^−1^ band intensity) as a function of the plasma activation time and the grafting process duration, respectively.

To characterize the chemical structure of the produced thin film more precisely, FTIR-ATR absorption measurements were performed. For the analysis, we adopted the band at 1650 cm^−1^, characteristic of N−H bonds in amines [41,42], which appears after the graft copolymerization of allylamine in a position where it does not interfere with the intense bands for the PET substrate (Figure 4). An example of the calibrated spectrum measured for such a sample in the region of this band and its peak fitting is shown in Figure 5a. Taking the intensity of the 1650 cm^−1^ band calculated on this basis, the dependences of the amount of allylamine grafted on the time of the plasma activation of the PET surface and the duration of the grafting process were determined, as shown in Figure 5b,c, respectively.

As can be seen in Figure 5b, with the time of plasma activation, there is initially an increase in the amount of allylamine grafted, reaching a maximum at about 120 s, and then decreasing for longer activation times. Such an effect, however, is justified by taking into account the dependence of the grafting process efficiency on the concentration of radical states generated on the surface. As the concentration of the radicals increases, they recombine more easily, which ultimately leads to a decrease in this concentration [43,44], and thus also to the lower activation of the grafting process. In turn, the analysis of the grafting time (Figure 5c) shows that the process is heading towards a termination state, which is probably mainly due to cross-linking between growing poly(allylamine) chains. For further studies, the plasma activation time equal to 120 s and the grafting time equal to 2 h were assumed as the optimal conditions for the preparation of the matrix from allylamine, which was deposited on the textiles in order to anchor the appropriate antifungal compounds.

**Figure 6:**
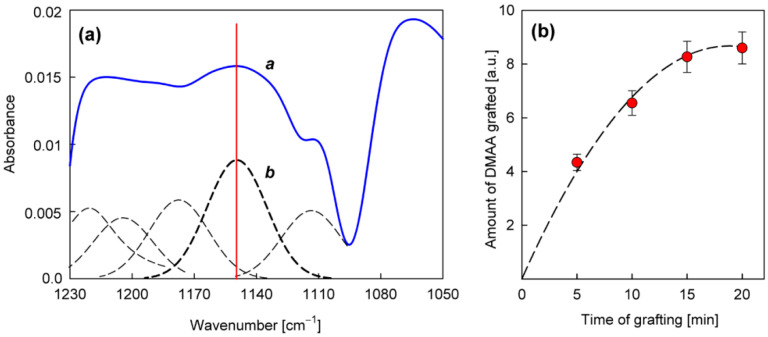
Characterization of the nanocoatings obtained from *N*,*N*-dimethylallylamine in plasma-activated grafting: (**a**)—An example of calibrated (*a*) and peak-fitted (*b*) FTIR-ATR spectrum in the region of 1150 cm^−1^ band (activation: Ar plasma, 40 W, 120 s; grafting: 15 min); (**b**)—The amount of *N*,*N*-dimethylallylamine grafted (calculated from the 1150 cm^−1^ band intensity) as a function of the grafting process duration.

The precursor selected from the group of amines for the direct production of the nanocoating with potential antifungal properties was *N*,*N*-dimethylallylamine (Table 3). The deposition process was carried out in the same way as in the case of allylamine, by plasma-activated grafting on the PET substrate. Argon plasma was used to activate the PET surface, assuming the same optimal parameters as for allylamine grafting (power 40 W, treatment time 120 s). Then, *N*,*N*-dimethylallylamine saturated vapor at room temperature (22.8 kPa) was introduced into the reactor chamber (without plasma) to perform the grafting process. The formation of nanocoatings was confirmed by FTIR-ATR analysis, where as a characteristic band, not interfering with the PET bands (Figure 4), we selected the band at 1150 cm^−1^ assigned to the stretching vibrations of C−N bonds in tertiary amines [41,42]. An example of a calibrated spectrum for the region of this band and its peak-fitting is shown in Figure 6a. The intensity of the 1150 cm^−1^ band served as a measure of the amount of *N*,*N*-dimethylallylamine grafted onto the PET substrate. Figure 6b shows the dependence of such a represented amount of nanocoating on the grafting time. The grafting process in this case reaches a saturation state much faster than with allylamine. Therefore, we assumed 15 min as the optimal time for fabric surface modification.

**Figure 7:**
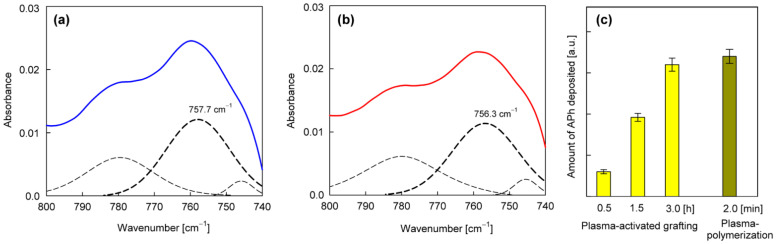
Characterization of the nanocoatings obtained from 2-allyphenol: (**a**,**b**)—Examples of calibrated and peak-fitted FTIR-ATR spectra in the region of 757 cm^−1^ band for 2 min plasma polymerization and 3 h grafting, respectively; (**c**)—A quantitative comparison of these two deposition processes of nanocoating from 2-allylphenol.

From the group of phenols (Table 3), 2-allylphenol was selected for further investigation. In this case, the antifungal nanocoatings were prepared in two ways, both starting with the initial plasma activation of the PET surface (Ar plasma, power 40 W, treatment time 120 s). In the first way, grafting of 2-allylphenol was performed without plasma at a temperature of 120 °C, a precursor flow rate of 3.0 sccm, and a pressure in the reactor chamber of about 100 Pa. In the second case, we used plasma generated with 5 W power in a mixture of argon and precursor with flow rates equal to 1.0 sccm and 0.3 sccm, respectively, at a total pressure in the reactor chamber of about 8.0 Pa. The deposition process was also carried out at a temperature of 120 °C, and its rate was approximately 0.5 nm/s (estimated from interferometric measurements). To characterize the deposited nanocoatings, an FTIR-ATR band at about 757 cm^−1^ (not interfering with the PET bands (Figure 4)) was chosen. This band is attributed to the C−H wagging vibration in an ortho-substituted benzene ring [41,42] and is typical for 2-allylphenol [45]. Figure 7a,b show examples of calibrated spectra in the region of the 757 cm^−1^ band and their peak fitting for such nanocoatings deposited with grafting (without plasma) and produced by plasma polymerization. In turn, the amount of nanocoating expressed as the intensity of the 757 cm^−1^ band for different grafting times (from 0.5 to 3 h) and for comparison, for 2 min plasma polymerization, is shown in Figure 7c.

As you can see, 2 min plasma polymerization allows us to produce even a slightly greater amount of the nanocoating than 3 h grafting, while maintaining practically the same chemical structure, as shown by the spectra in Figure 7a,b. Therefore, only the plasma polymerization method of 2-allylphenol was chosen for the further surface modification of the fabrics.

### 3.3. Antifungal Activity of Nanocoatings on Textiles

Testing of the antifungal properties of the nanocoatings produced on the textiles was carried out for their four types listed in Table 1 and for four selected species of mold (Section 2.2), using the following surface preparation procedures based on the above-presented studies on samples with PET substrate (see Figure 1):

M-0–Textiles without any antifungal nanocoatings;M-DMAA–The nanocoating prepared by plasma-activated grafting of *N*,*N*-dimethylallylamine (plasma activation: Ar, 40 W, 120 s; grafting: saturated vapor of 22.8 kPa, 15 min);M-AA+DCP–Nanocoating prepared by plasma-activated grafting of allylamine (plasma activation: Ar, 40 W, 120 s; grafting: saturated vapor of 32.3 kPa, 2 h), and then anchoring 3,5-dichlorophenol (Section 2.4);M-AA+TCS–Nanocoating prepared by plasma-activated grafting of allylamine (plasma activation: Ar, 40 W, 120 s; grafting: saturated vapor of 32.3 kPa, 2 h), and then anchoring triclosan (Section 2.4);M-APh–Nanocoating prepared by plasma polymerization of 2-allylphenol (plasma activation: Ar, 40 W, 120 s; plasma polymerization: mixture of Ar and 2-allylphenol with flow rates of 1.0 and 0.3 sccm, respectively, 8.0 Pa, 5 W, 120 °C, 2 min).

**Figure 8:**
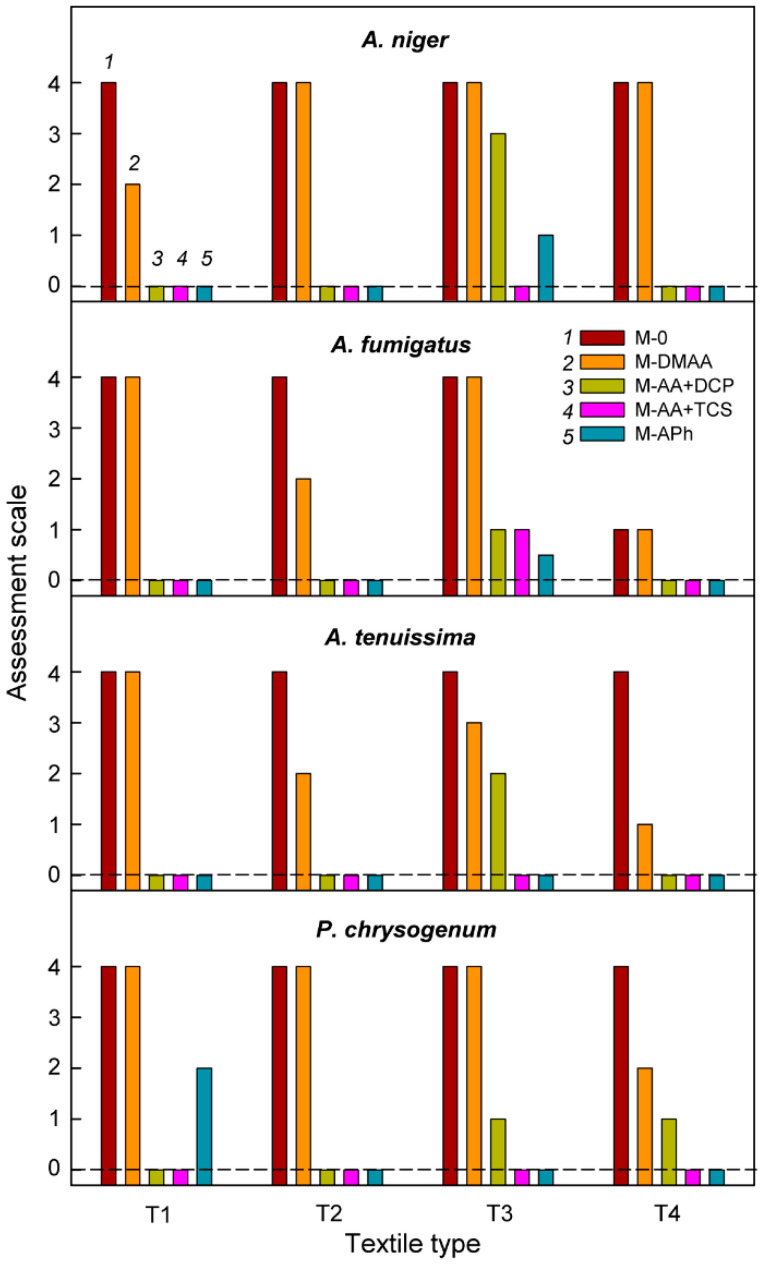
Antifungal activity of the surface of textiles (T1–T4) depending on the type of nanocoating (*1*–*5*) and the species of mold fungi. The nanocoating types in each case are provided in the same order as for *A. niger* and textile T1.

Figure 8 shows the dependence of the antifungal activity of the textile surface, expressed on an assessment scale (Section 2.6), as a function of the type of nanocoating and the species of mold fungi. The applied long incubation period (14 days) allows for unequivocal determination of the antifungal activity of nanocoatings in comparison with the surfaces not subjected to modification.

As can be seen, the M-DMAA nanocoating has the worst performance. In contrast, the other three modifications generally work very well, leading to minimal mold growth only in a few cases. Particularly noteworthy here is the M-APh nanocoating, which is strongly bonded to the textile surface and as a covalent macromolecular material, cannot diffuse into the environment, so it is much more durable than nanocoatings, such as M-AA+DCP and M-AA+TCS, in which we anchored molecules capable of slowly diffusing out of the matrix. Very good antifungal properties, which ensured the durability of the nanocoating, the originality of the solution based on the plasma polymerization process under mild conditions allowing to keep intact fragments of the molecule responsible for its antifungal nature, and of course, the large application prospects associated with it, provided grounds for granting a patent for the plasma deposition of antifungal structures from 2-allylphenol on textile surfaces [46].

**Figure 9:**
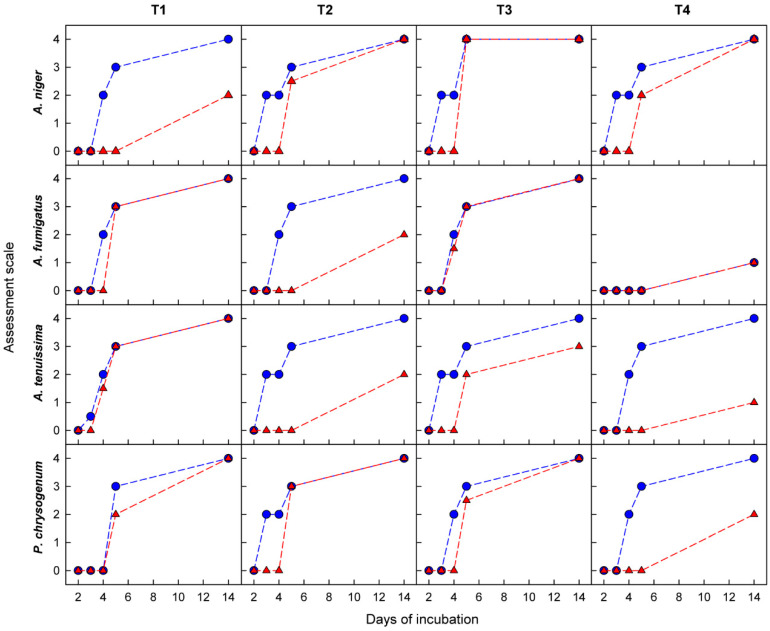
Antifungal activity of the surface of textiles (T1–T4), bare (blue circles) and covered with the M-DMAA nanocoating (red triangles), depending on the incubation period and the mold fungi species.

Returning to the M-DMAA nanocoating, which reveals a very weak antifungal activity on textile surfaces, in contrast to the behavior of the isolated *N*,*N*-dimethylallylamine precursor (Table 3), the activity of this nanocoating as a function of the incubation period was investigated. Such relationships for the tested textiles and species of mold fungi are shown in Figure 9. In virtually every case studied, the nanocoating in the initial period shows a significant antifungal activity compared to the bare textiles, but then, with the incubation time, it clearly decreases, often disappearing completely. These observations, relying on findings regarding the mechanism of action of amine biocides, which consist in damaging the cell membrane [8,10], lead to the conclusion that, probably as a result of the interaction of the nanocoating amino groups with the just damaged fungal cell, these groups are stably bonded with unspecified cytosolic components, which limits their further antifungal activity.

## 4. Conclusions

The results presented in this paper reinforce the belief that cold plasma technology plays a significant role in the practical modification of surfaces, and in this particular case, they demonstrate its successful application in the production of anti-mold nanocoatings on textiles. We tested three different procedures to obtain such nanocoatings: (i) plasma-activated graft copolymerization as an active antifungal modification in itself, (ii) matrix preparation by plasma-activated graft copolymerization and anchoring biocidal molecules therein, and (iii) most promising, both in terms of technology, durability, and activity of the nanocoating—plasma polymerization of the precursor with biocidal properties, performed in such a way as not to destroy these properties. In the latter case, the chemical structure of the precursor molecule is of crucial importance. It must consist of two elements: one of them is responsible for the biocidal properties, while the other is involved in the plasma polymerization process. The first of these elements is adequately resistant to plasma, and the second, in turn, is decomposed in plasma, enabling the chemical incorporation of the molecule into the structure of the created plasma polymer film. Using a molecule constructed in this way and selecting the appropriate conditions for the plasma polymerization process, we obtained a solid nanocoating with a typical thickness of several dozen nanometers, showing biocidal activity. In fact, this technologically simple, short-duration, and practically waste-free plasma polymerization process, whose advantage is also the possibility of using many different molecular structures of precursors, is a very promising solution for the further search for more and more efficient and useful antifungal nanocoatings for textiles.

## Figures and Tables

**Table 1:** Textile fabrics selected for testing.

Textile	Type of Material
T1	Micro Rip-Stop 100% polyester (both sides)
T2	Micro Rip-Stop inner side—100% polyester outer side—100% polyurethane
T3	100% natural cotton (both sides) grammage 165 g/m^2^
T4	Micro Rip-Stop inner side—100% nylon outer side—100% silicon

**Table 2:** Precursors used in the production of anti-mold nanocoatings.

Precursor	Formula	Characteristic
**Phenols**		
2-Allylphenol (**APh**)	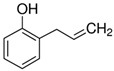	liquid assay 98% b.p. 220 °C
2-Methoxy-4-(2-propenyl)phenol (Eugenol)	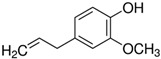	liquid assay 99% b.p. 254 °C
**Amines**		
Allylamine (**AA**)	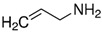	liquid assay 98% b.p. 53 °C
*N*,*N*-Dimethylallylamine (**DMAA**)	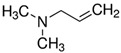	liquid assay ≥ 98% b.p. 64 °C
3-Dimethylamino-1-propyne	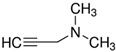	liquid assay 97% b.p. 81 °C
Diallyl-dimethylamonium chloride	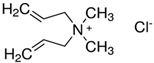	65 wt.% in H_2_O
**Anchored Compounds**		
4-Chloro-2-isopropyl-5- methylphenol	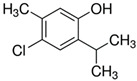	crystals assay 99% ethanol: soluble 10%
3,5-Dichlorophenol (**DCP**)	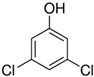	crystals assay 97% ethanol: very soluble
2-Isopropyl-5-methylphenol (Thymol)	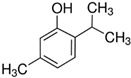	crystals assay ≥ 98.5% ethanol: soluble 5%
5-Chloro-2-(2,4-dichlorophenoxy) phenol (Triclosan) (**TCS**)	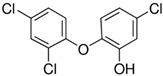	crystals assay 99% ethanol: very soluble

**Table 3:** Assessment of the antifungal activity of the precursors from Table 2 in relation to the selected species of mold fungi.

	Diameter * of The Growth Inhibition Zones [mm]							
Mold Fungi Species →	*A. niger*		*A. fumigatus*		*A. tenuissima*		*P. chrysogenum*	
**Precursor concentration [mol/L]** **→**	0.01	0.1	0.01	0.1	0.01	0.1	0.01	0.1
**Incubation time [h]** **→**	24	24	24	24	72	72	72	72
**Precursor type** **↓**								
2-Allylphenol **(APh)**	18	50	10	41	10	56	0	40
2-Methoxy-4-(2-propenyl)phenol (Eugenol)	14	43	13	33	10	60	10	37
Allylamine **(AA)**	0	0	0	0	0	0	0	0
*N*,*N*-Dimethylallylamine (**DMAA**)	8	35	7	27	17	47	0	34
3-Dimethylamino-1-propyne	8	16	0	7	10	14	0	10
Diallyl-dimethylamonium chloride	0	0	0	0	0	0	0	0
4-Chloro-2-isopropyl-5-methyl-phenol	12	28	13	32	18	49	7	15
3,5-Dichlorophenol (**DCP**)	26	52	18	58	49	82	33	72
2-Isopropyl-5-methyl- phenol (Thymol)	16	62	14	49	14	49	0	22
5-Chloro-2-(2,4-dichlorophenoxy) phenol (Triclosan) **(TCS)**	20	22	22	23	42	44	38	40

* This value is expressed as the difference between the diameter of the inhibition zone and the diameter of the hole in the medium (8 mm).

## Data Availability

The data presented in this study are available upon reasonable request from the corresponding author.
